# Wide-field corneal subbasal nerve plexus mosaics in age-controlled healthy and type 2 diabetes populations

**DOI:** 10.1038/sdata.2018.75

**Published:** 2018-04-24

**Authors:** Neil S. Lagali, Stephan Allgeier, Pedro Guimarães, Reza A. Badian, Alfredo Ruggeri, Bernd Köhler, Tor Paaske Utheim, Beatrice Peebo, Magnus Peterson, Lars B. Dahlin, Olov Rolandsson

**Affiliations:** 1 Department of Ophthalmology, Institute for Clinical and Experimental Medicine, Linköping University, 581 83 Linköping, Sweden; 2 Institute for Applied Computer Science, Karlsruhe Institute of Technology, 76131 Karlsruhe, Germany; 3 Department of Information Engineering, University of Padova, 35122 Padova, Italy; 4 Faculty of Health Sciences, University College of Southeast Norway, 3045 Drammen, Norway; 5 Unit of Regenerative Medicine, Department of Medical Biochemistry, Oslo University Hospital; University of Oslo, 0407 Oslo, Norway; 6 Department of Public Health and Caring Sciences, Section of Family Medicine and Preventive Medicine, Uppsala University, 751 22 Uppsala, Sweden; 7 Department of Translational Medicine - Hand Surgery, Lund University, Skåne University Hospital, 222 41 Malmö, Sweden; 8 Department of Public Health and Clinical Medicine, Section of Family Medicine, Umeå University, 901 87 Umeå, Sweden

**Keywords:** Diagnostic markers, Type 2 diabetes, Autonomic nervous system, Eye manifestations, Neurological manifestations

## Abstract

A dense nerve plexus in the clear outer window of the eye, the cornea, can be imaged *in vivo* to enable non-invasive monitoring of peripheral nerve degeneration in diabetes. However, a limited field of view of corneal nerves, operator-dependent image quality, and subjective image sampling methods have led to difficulty in establishing robust diagnostic measures relating to the progression of diabetes and its complications. Here, we use machine-based algorithms to provide wide-area mosaics of the cornea’s subbasal nerve plexus (SBP) also accounting for depth (axial) fluctuation of the plexus. Degradation of the SBP with age has been mitigated as a confounding factor by providing a dataset comprising healthy and type 2 diabetes subjects of the same age. To maximize reuse, the dataset includes bilateral eye data, associated clinical parameters, and machine-generated SBP nerve density values obtained through automatic segmentation and nerve tracing algorithms. The dataset can be used to examine nerve degradation patterns to develop tools to non-invasively monitor diabetes progression while avoiding narrow-field imaging and image selection biases.

## Background and Summary

In recent years there has been a growing interest in using the cornea as a window into the body, to develop early and sensitive biomarkers of disease. The cornea in particular is richly innervated by unmyelinated axons (C-fibers) of the peripheral nervous system, which among other important functions release growth factors and stimulate the eye’s protective blink reflex^1^. Within the cornea, peripheral nerves form a dense subbasal nerve plexus (SBP) layer that can be visualized by a non-invasive clinical examination termed *in vivo* confocal microscopy (IVCM) or corneal confocal microscopy (CCM)^2–5^. Using IVCM, high-resolution two-dimensional images of the living SBP can be acquired and characterized. Corneal nerve fiber length density (CNFL), a putative biomarker of peripheral nerve fiber degeneration, has been associated with a range of diseases including ocular pathologies^6–9^, cancer^10^, and neurodegenerative disease such as amyotrophic lateral sclerosis^11^, Parkinson’s disease^12^, and type 1 (refs 13–18) and type 2 (refs 17–20) diabetes and its complications, such as peripheral diabetic neuropathy^13–20^, diabetic retinopathy^21–24^, cardiac autonomic neuropathy^13,14,25^, and diabetic nephropathy^26^. These studies demonstrate the potential of IVCM to directly monitor peripheral nerve degeneration *in vivo*, which is not feasible using existing invasive methods such as intra-epidermal peripheral nerve measurement by skin biopsy.

Several obstacles, however, prevent reliable and accurate assessment of corneal nerves. IVCM provides narrow-field images, each depicting an exceedingly small area (less than 1%) of the central cornea. Multiple images from the SBP are therefore typically sampled for analysis^27,28^, an approach that is subjective, non-standardized, prone to image selection bias and has poor reproducibility^29,30^. Producing wide-field images of the SBP, however, is technically challenging and the time required for wide-field image acquisition, mosaic reconstruction, and nerve analysis has been too lengthy to be applicable in a clinical setting^19^. Compounding this problem, nerve paths in the SBP vary in three dimensions due to the corneal curvature and local tissue variations, and 2D confocal image slices in a fixed plane do not capture this variation; the magnitude of the resulting error is unknown^19^. Because of these multiple difficulties, a large variability in methodology and in corneal nerve parameters has been reported in prior studies^31,32^. These significant obstacles, however, must be overcome in order to standardize IVCM for routine clinical use and provide a non-invasive tool for screening and assessment of corneal nerves of the SBP.

In this study, the aim was to produce a dataset of high-quality wide-field images of the human corneal SBP, to be used for analysis of signs or patterns of peripheral neurodegeneration. To minimize human sampling biases, wide-field images were constructed from individual microscopic fields of view that were machine-assembled into mosaics with an optimized algorithm projecting raw acquired 3D data onto a two-dimensional plane for maximum visibility of nerve paths. Nerve quantification bias was minimized by applying an automated nerve tracing and quantification algorithm to yield SBP nerve density values, which was validated by comparison with manual nerve tracing analysis. Inter-eye comparisons within the same subject are made possible by bilateral mosaics provided in the dataset, and age is mitigated as a confounding factor by providing mosaics representing a group of same-aged subjects. Finally, the examined cohort of subjects comprised healthy, impaired glucose tolerance, short-duration and long-duration type 2 diabetes groups, to enable analyses of SBP parameters with development and progression of type 2 diabetes.

The dataset has extensive reuse potential. Intra- and inter-subject variations in the SBP can be examined and related to the provided clinical parameters. Although we provide quantitative data for nerve density, other parameters can be analyzed (such as nerve number, type, branching, tortuosity, sprouting, degradation, etc.) and related to duration of diabetes, blood HbA1c level, body mass index, etc. The dataset also depicts the infero-central ‘whorl’ region within the SBP and we provide an illustrative quantification of this region, but further analyses and definitions of the whorl are possible. The SBP is the site of inflammatory dendritic cell accumulation in the cornea^4^, and these cells in the dataset could be quantified for possible relation to disease. Finally, the anatomy of the human SBP in a wide field of view has not previously been studied in a large group of subjects, and the provided dataset could yield new insights into this important anatomic layer of the cornea that is linked to systemic neurodegenerative disease.

## Methods

### Clinical study design and subjects

A full description of the cohort of subjects with inclusion and exclusion criteria has been given elsewhere^33^. Subjects were examined based on an initial cohort of 129 age- and sex-matched subjects aged 60±1 years at initial inclusion in 2004 (ref. 34). At a 10-year follow-up examination in 2014, ophthalmic examination was conducted in all evaluable subjects at the Eye Clinic of the Skellefteå Hospital, Sweden. All subjects gave written informed consent to participate and the protocol was approved by the ethical review board of the University of Umeå, Umeå, Sweden (Ethical application no. 2013-21-31M), complying with the tenets of the Declaration of Helsinki.

### Ophthalmic examinations

Bilateral corneal examination of study subjects included IVCM assessment of the SBP (Heidelberg Retinal Tomograph 3 with Rostock Cornea Module, Heidelberg Engineering, Germany) by a single examiner, and contact esthesiometry (Cochet-Bonnet contact esthesiometer, Luneau Technologies, France) by a different single examiner. Examiners were masked to the health status of the subjects and result of other ophthalmic examinations at the time of examination.

A total of 164 eyes from 82 subjects were examined. All eye examinations were completed during a five-day period in an ophthalmology outpatient clinic, representing about 32 eyes per day. The cohort was controlled in age to minimize possible age-related effects (69.1±1.2 years, mean±SD) and all subjects resided in the same county in Northern Sweden for at least 10 years prior to examination.

### Adaptive method for depth-corrected SBP mapping by CCM

Routine clinical acquisition of wide-field mosaics of the corneal SBP that account for microscopic deviation of nerve paths in the axial direction is a significant technical challenge. Optimal focus for a single field (area 0.16 mm^2^) can be found, but translation of the field of view to obtain wide-field imaging requires real-time depth correction. An approach of acquiring full 3D confocal image stacks from adjacent fields of view and projecting and stitching these into a 2D mosaic has been proposed, but this technique rapidly increases clinical examination time and only small mosaics representing 3-4 fields of view are feasible^19^. Likewise, an automated fast-acquisition technique to scan a larger area of the SBP has been recently reported^35^, but was limited to single-depth image acquisition. To overcome these limitations, an adaptive examination method was employed combining raster translation of the field of view laterally (in the xy-plane parallel to the corneal surface) with real-time manual axial depth correction (z-axis perpendicular to the corneal surface) across a limited axial range, to obtain small confocal image stacks of 2–5 images. The procedure was implemented while the subject maintained a fixed gaze towards a built-in white light diode target. Raster scanning was by manual x and y translation of the microscope objective to sweep over the visible area of the SBP while depth correction was adaptively implemented to capture the full three-dimensional variation of subbasal nerve paths. To achieve this, the focal plane was adjusted by several microns anteriorly and posteriorly in a quick anterior-to-posterior motion while corneal nerves were visible in real time on a computer screen, using a motorized joystick attachment set to the fastest axial speed. The resulting depth information was represented in an image stack containing 2 to 5 confocal image slices (with axial spacing of 0.5–2 μm), which is sufficient to capture axial nerve path variation within the thin SBP. To permit non-central areas of the SBP to be included (including the infero-central whorl region), the entire adaptive scan procedure (raster scan with axial adjustments) was repeated four more times, after moving the fixation target slightly towards superior, inferior, nasal, and temporal directions but maintaining a predominantly central and paracentral corneal location. Throughout the acquisition process, image data was recorded at a rate of 8 images per second. Following image acquisition and removal of images not in focus and not containing the SBP (Data Citation 1), images were subjected to further processing and analysis.

### Automated mosaicking of IVCM image data

The mosaicking process is an improvement of a prior method^36^ used to create projected images from IVCM volume scans^37^ and two-dimensional mosaics of the SBP from temporally consecutive IVCM sequence scans^35^. Briefly, the original mosaicking algorithm can be described as follows. Let [*I*_*i*_] (1≤*i*≤*N*) be the acquired sequence of *N* images, each with a size of 384×384 pixels. In short, the core of the process consists of decomposing each *I*_*i*_ into 12 equally-sized horizontal stripes *S*_(*i*,1)_, …, *S*_(*i*,12)_ and aligning these sub-images for selected pairs of images (*I*_*i*_,*I*_*j*_) using the phase correlation function. This sub-image approach ensures reliable alignment results and facilitates good estimation of the characteristic, motion-induced distortion patterns present in the image data. After validating the correlation results by comparison of the correlation values with a predefined threshold, each successful sub-image registration yields a translation vector ***u***_(*i*,*m*),(*j*,*n*)_ (1≤*m*,*n*≤12), that equals the difference vector ***p***_(*j*,*n*)_−***p***_(*i*,*m*)_=***u***_(*i*,*m*),(*j*,*n*)_ of the (unknown) positions ***p***_(*i*,*m*)_ and ***p***_(*j*,*n*)_ of *S*_(*i*,*m*)_ and *S*_(*j*,*n*)_, respectively, in the mosaic image. The system that arises from the linear equations given above can be solved for the sub-image positions using the least squares approach; however, as the system matrix is always close to singular, additional regularization constraints have to be introduced. Once all of the estimated sub-image position coordinates ***p***_(*i*,1)_, …, ***p***_(*i*,12)_ in the mosaic image coordinate system are known, they are interpreted as sampling points along a smooth curve which describes the position coordinates for all 384 pixel rows of all images of the sequence and which is approximated by an interpolating spline function through the ***p***_(*i*,*m*)_. Transformation of the original image rows of an image *I*_*i*_ to their appropriate destination positions in the mosaic image coordinate system provides the motion-corrected (and correctly positioned) transformed image *I*_*i*_’. A more detailed description of the image registration process can be found elsewhere^36^. The mosaic image is finally assembled by weighted averaging of all *I*_*i*_’ as described previously^38^.

The process as used in the present study differs from that in previously published work in two ways. The first difference was that images constituting a dataset in this study were acquired in multiple sequence scans containing small volume stack data (not full IVCM volume scans^19^) with subsequent manual exclusion of incorrectly focused images. This was done to significantly speed the examination time while greatly expanding the imaged area and simultaneously including depth-dependent information. Images within an acquired sequence scan were not considered to represent a continuous image sequence but regarded to be acquired independently. No underlying pattern was assumed with respect to the relative image positions, and selection of image pairs to be aligned included all *N*(*N*-1)/2 possible image pairs (*I*_*i*_,*I*_*j*_) with 1≤*i*<*j*≤*N*. The second difference was with regard to the regularization of the system of equations. Instead of constraining the smoothness of the curve on which the sub-image positions lie across the entire image sequence, the curve was partitioned into *N* subsections, each corresponding to a single image, and the smoothness constraint was applied to each subsection independently. All mosaic images were generated fully automatically without human intervention.

### Characteristics of wide-field depth-corrected mosaics

**Figure 1:**
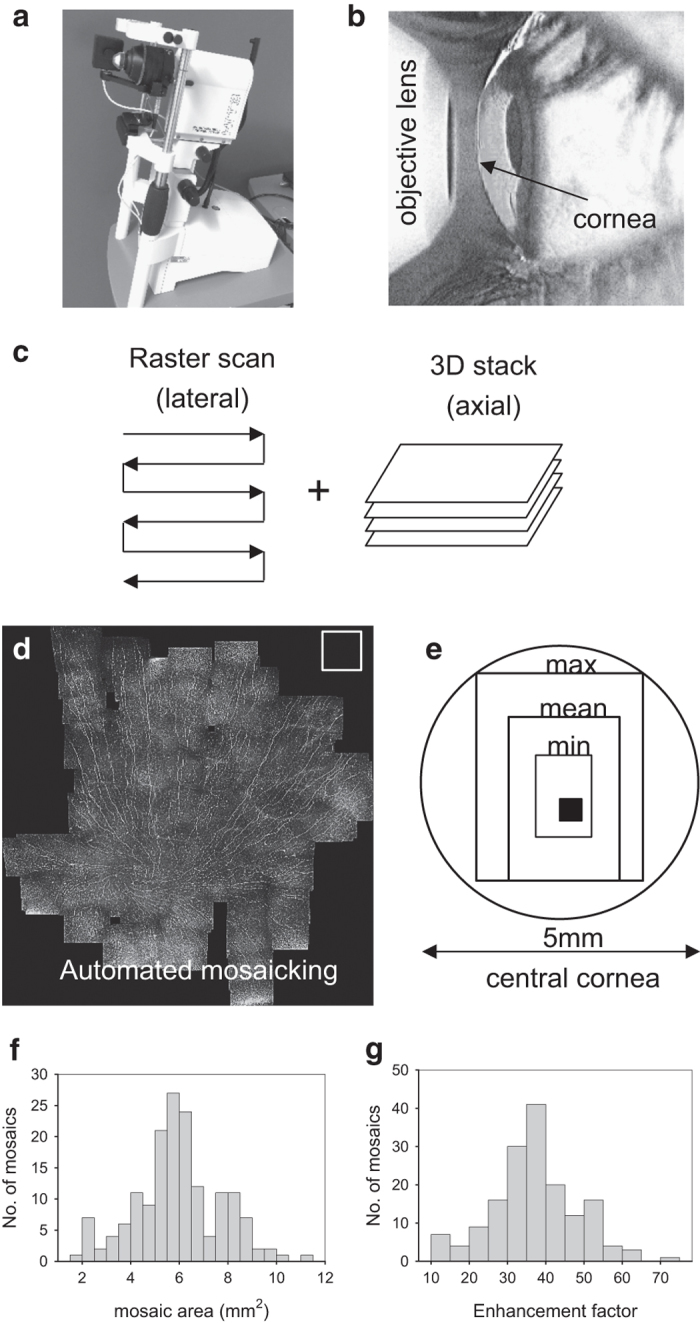
Method for obtaining wide-area depictions of the corneal SBP by obtaining 3D image data *in vivo* and applying an automated mosaicking algorithm. (**a**) Laser-scanning *in vivo* corneal confocal microscope used to obtain images of the SBP. (**b**) The cornea is scanned by placing the microscope objective lens in contact with the cornea, using a drop of transparent ophthalmic gel for refractive index matching and physical coupling (not shown). (**c**) Adaptive method combining manual raster scanning with real-time manual depth-correction in the axial direction to create small confocal image stacks of 2-5 images. (**d**) Automated processing of raw image sets to produce wide-field mosaics. The white box in the top right corner of the mosaic represents the size of a single field of view of the microscope, 400×400 μm^2^. (**e**) The range of mosaic areas obtained in the present dataset represents a significant proportion of the central cornea compared to a single field of view (filled black square). (**f**) Histogram of mosaic size in the dataset, and (**g**) histogram of area enhancement factor relative to a single field of view, for the 163 mosaics comprising the dataset.

IVCM examinations were conducted with a typical clinical examination time of five minutes per eye (Fig. 1a,b). Using an adaptive imaging method (Fig. 1c) the cornea was manually scanned in a raster pattern to achieve wide lateral coverage of the SBP while adaptively controlling focus (axial depth) in real time to capture the best focal plane of nerve paths. This consisted of adjusting focal depth during image acquisition to achieve small confocal image stacks of 2–5 images per field of view, to essentially capture the entire depth variation of nerves in the thin SBP. The acquisition method was implemented on standard commercially-available IVCM equipment, without hardware or software modifications or upgrades. Using a post-acquisition mosaicking algorithm, the raw images were automatically assembled into wide-field mosaics, with a representative mosaic indicated in Fig. 1d. A single field-of-view image size is indicated (white square, Fig. 1d) along with the relative proportion of the central cornea captured by the adaptive approach in the present study (Fig. 1e). Using raw images obtained by the adaptive IVCM method, mosaics were constructed in 164 eyes from 82 subjects in the cohort (100% success rate), with mosaic size and processing time given in Table 1. In one eye, however, IVCM image data was not of sufficiently high quality to achieve a good, wide-field representation of nerves in the subbasal plexus. In this single case (left eye of subject ID 70), large gaps in the nerve plexus were apparent due to missing image data in the plexus layer (IVCM images were either out-of-focus or in the plane of epithelial cells). For the single largest mosaic per eye, mean depth-corrected mosaic area was 5.95 mm^2^ across 163 mosaics (Fig. 1f) corresponding to a mean enhancement factor of 37 (Fig. 1g) compared to a single microscope field of view. A mean of 522 raw images were used to construct a mosaic, with a mean processing time of 106 min per mosaic. Subsequent optimization of the implementation of the mosaicking algorithm improved processing time by a factor of 15. The optimized algorithm yielded a processing time of 7 min for an average-sized mosaic. In total 322 mosaics were produced (Data Citation 1). The image alignment and mosaicking algorithms were implemented in C++, and all runtimes were measured on a Windows PC system (Core2 Duo E8400, 2×3 GHz, 6 GB RAM).

### Nerve tracing and analysis of data usability and quality

Nerve fibers visible in all mosaics were traced using two approaches: manual and fully automated. An experienced observer manually traced the centerlines of all visible nerves using the NeuronJ tracing plugin for ImageJ (publicly available at http://imagej.nih.gov/ij/) in the acquired images, as previously described^39^. The resulting manual tracings were checked for consistency and accuracy by a second, independent experienced grader, and anomalies and missed nerves in the original manual tracings were corrected. The total length of all traced nerves was determined in NeuronJ in millimeters, and this value was divided by the mosaic area in mm^2^, yielding the mosaic nerve fiber length density (mCNFL) in mm/mm^2^ (Data Citation 1).

Automatic nerve tracing was based on a previously developed algorithm^40^, but applied here to large-scale mosaics for the first time. Briefly, corneal nerve visibility was initially enhanced using a bank of log-Gabor filters. The resulting filtered image was then thresholded to obtain candidate nerve segments (all string-like structures present in the image). Finally, these candidate nerve segments were classified as nerves or 'other' using a Support Vector Machines (SVM) approach. The length of all traced segments was then automatically determined, and divided by the mosaic area to yield mCNFL in mm/mm^2^ (Data Citation 1). Specifically for mosaics, to avoid underestimation bias due to mosaic edge effects (where no nerves are visible due to partial oblique imaging of the corneal epithelium or Bowman’s layer), the mosaic area used for the nerve length density calculation excluded these edge regions containing anatomic layers outside the SBP (for both manual and automatic approaches).

To demonstrate the possibility to individually analyze specific regions of the SBP, nerve length density within the infero-central whorl^26,41,42^ region (wCNFL) was computed. An expert identified the center position (origin) of the whorl region in each mosaic containing an identifiable whorl. Then a circular region with 800 μm diameter and centered on the origin was defined. Nerve length density within this region was computed where full image data was available (Data Citation 1).

All mosaics were computer-analyzed and investigators at the site where nerve tracing and quantification was performed (University of Padova) were masked to the identity of subjects and subject groups.

### Statistical analysis

Correlation of mCNFL with nerve analysis method, between eyes of the same subject, and with esthesiometry values was assessed by Pearson correlation. Statistics were performed using SigmaStat 3.5 for Windows (Systat Software Inc., Chicago, USA), with a two-tailed value of α<0.05 considered as significant.

### Code availability

The computer code used to generate the depth-corrected mosaics reported in the study and the code used for automated nerve tracing were developed at academic institutions (Karlsruhe Institute of Technology and University of Padua, repectively) and are intended solely for scientific research. The developers of the code are willing to apply the algorithms to data sets provided by the research community through academic collaboration. Interested readers should contact authors Allgeier (mosaicking) and Ruggeri (nerve tracing) for further details.

## Data Records

Mosaic images of the corneal SBP acquired in vivo using laser-scanning confocal microscopy of the cornea in healthy subjects and type 2 diabetes mellitus subjects of the same age are provided (Data Citation 1). Mosaics represent wide-field images of the plexus incorporating 3D nerve path data projected onto a 2D plane. Images are in TIFF file format, labelled with the study subject ID number (number from 1 to 128), eye (OD for right eye, OS for left eye), and mosaic number, since data from a single eye often consisted of more than one mosaic (separate mosaics are given where image data did not overlap and could therefore not be stitched together in a single mosaic). In total 322 mosaics (each a separate TIFF image file) are provided, representing bilateral eye data from 82 subjects.

An Excel file (Data Citation 1) is also provided as a ‘key’ to the mosaic dataset. The Excel file contains detailed information indexed to the name of each mosaic image (for the largest mosaic per eye), including subject ID number, eye, mosaic area (effective area depicting the SBP, excluding portions of the mosaic not including the SBP), clinical esthesiometry data (cm of Cochet-Bonnet esthesiometer thread length to elicit a blink response), mCNFL by manual and automated nerve tracing methods, wCNFL by manual and automated nerve tracing methods, diabetes status of each subject (NGT, normal gluocose tolerance; IGT, impaired glucose tolerance; and number of years since diagnosis of type 2 diabetes mellitus). Note that the groups NGT and IGT represent non-diabetes control subjects. This data is described in Table 2.

The following clinical/demographic parameters have also been included for each subject, indexed according to subject ID number: age, sex, smoking status (0=nonsmoker, 1=smoker), blood HbA1c level (mmol/mol), body mass index (kg/m^2^), average fasting plasma glucose (mmol/l) and average 2-hour plasma glucose (mmol/l). Table 3 describes this data.

## Technical Validation

### Validation of fully-automated nerve tracing and nerve density analysis in wide-field mosaics

**Figure 2:**
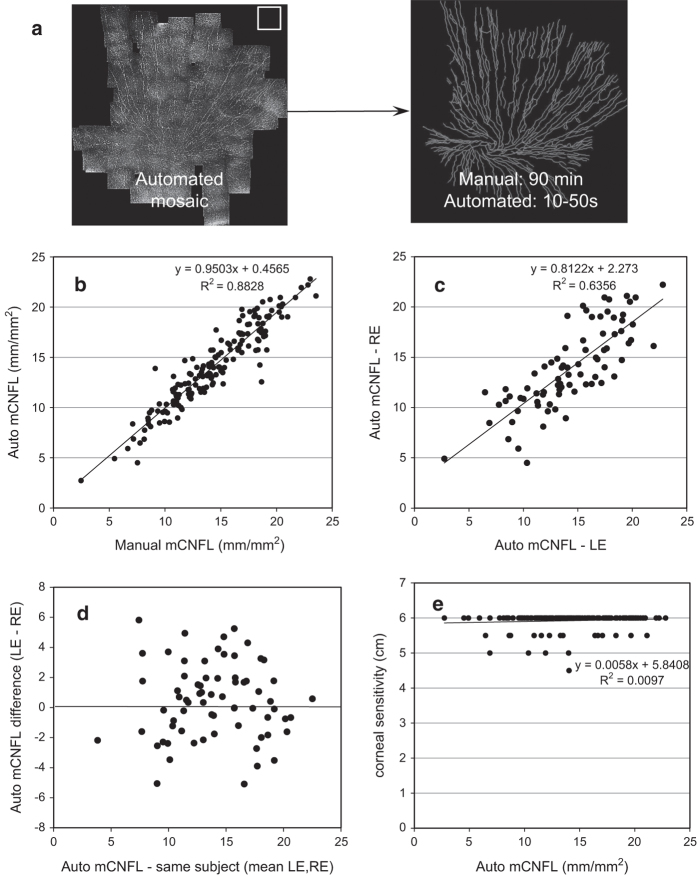
Validation of nerve tracing in SBP mosaics by manual and fully automated methods. (**a**) Tracing was completed manually and by a fully automated algorithm, with 100-fold improvement in speed by the automated method. (**b**) High correlation and linearity was evident by manual and automated methods. (**c**) Left eye (LE) and right eye (RE) values of mCNFL were highly linearly correlated. (**d**) mCNFL difference between eyes of the same individual indicates the degree of within-subject variability in the dataset. (**e**) Poor correlation of mCNFL with ocular surface sensitivity measured by clinical contact esthesiometry (in 5mm steps). In (**c**–**e**), results from manual tracing were nearly identical (not shown).

Manual tracing of nerve paths in the largest mosaic for each eye was completed by a trained human observer using NeuronJ to aid in semi-automatic nerve path detection^39^, with a resulting mean tracing time of 90 to 120 min per mosaic. Several weeks were required to manually trace all 163 mosaics. Fully-automated nerve tracing of the same mosaics was completed with processing times ranging from 10 to 50 s per mosaic, corresponding to a 100-fold improvement in speed relative to manual tracing (Fig. 2a). Agreement and linearity in the resulting mCNFL between manual and automated tracing methods was strong (Fig. 2b), with a Pearson correlation coefficient of r=0.94 (*P*<0.001). Mean difference in mCNFL between methods was 0.26±1.38 mm/mm^2^ and Bland-Altman 95% limits of agreement (LOA) were±2.71 mm/mm^2^ around the mean. Bilateral analysis (Fig. 2c) yielded a Pearson correlation of r=0.76 and 0.80 for mCNFL between left and right eyes of the same subject, for manual and automated analyses, respectively (both *P*<0.001). The mean difference in mCNFL between both eyes of the same subject was 0.3±2.71 mm/mm^2^ (manual) and 0.2±2.46 mm/mm^2^ (automated), with the variation in this difference depicted in Fig. 2d. The correlation of mCNFL with ocular surface sensitivity measured by clinical contact esthesiometry was assessed (Fig. 2e), with no correlation with esthesiometry values noted for either manual (r=0.06, *P*=0.48) or automated (r=0.10, *P*=0.21) tracing methods.

### Analysis of bias in CNFL estimates versus mosaic-based mCNFL

To evaluate the quality and usability of the mosaic depictions of the SBP relative to single raw image sampling without automated mosaicking or without post-processing for correcting nerve path variations in depth, a comparison between single images and mosaics was performed for two scenarios. The scenarios highlight the biases inherent in human subjective sampling of single IVCM images for analysis, versus use of depth-corrected mosaics. In Scenario 1 (sampling with correction for axial nerve path variation), non-overlapping 400×400 μm^2^ square image regions (the size of a single field of view image obtained during an IVCM examination) were cropped from each depth-corrected mosaic; region locations were the exact locations of single raw images of the acquired data. The resulting mean CNFL of these cropped frames was considered. In Scenario 2 (sampling of raw non-depth-corrected images), single raw IVCM images were taken from the same non-overlapping regions identified in Scenario 1. For both scenarios and 1 to 20 single non-overlapping IVCM images, mean CNFL was calculated for each possible combination of the selection (for example, if 3 non-overlapping IVCM images are selected per eye, there are 1140 possible ways to choose these 3 fields among a total of 20 images). Mean CNFL was then compared to the reference mCNFL to determine the relative error (% error referenced to mCNFL).

**Figure 3:**
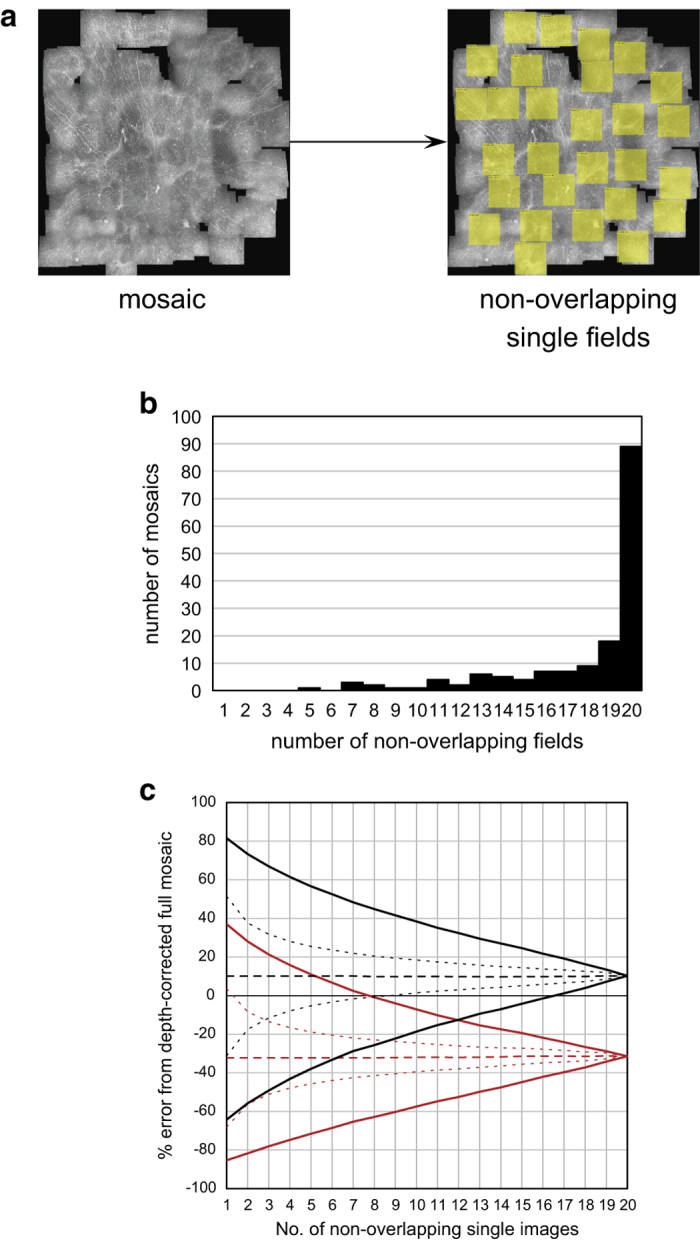
Analysis of bias from sampling single IVCM images to estimate CNFL versus wide-field mosaic mCNFL in 160 mosaics. (**a**) Each mosaic was deconstructed into 20 non-overlapping single image fields (actual image locations). (**b**) Histogram of the number of non-overlapping fields per mosaic. 107 out of 160 mosaics had at least 19 non-overlapping fields. (**c**) Error analysis in Scenario 1 with depth correction (black; mean=dashed, standard deviation=dotted, range=solid) and Scenario 2 without depth correction (red; mean=dashed, standard deviation=dotted, range=solid).

**Figure 4:**
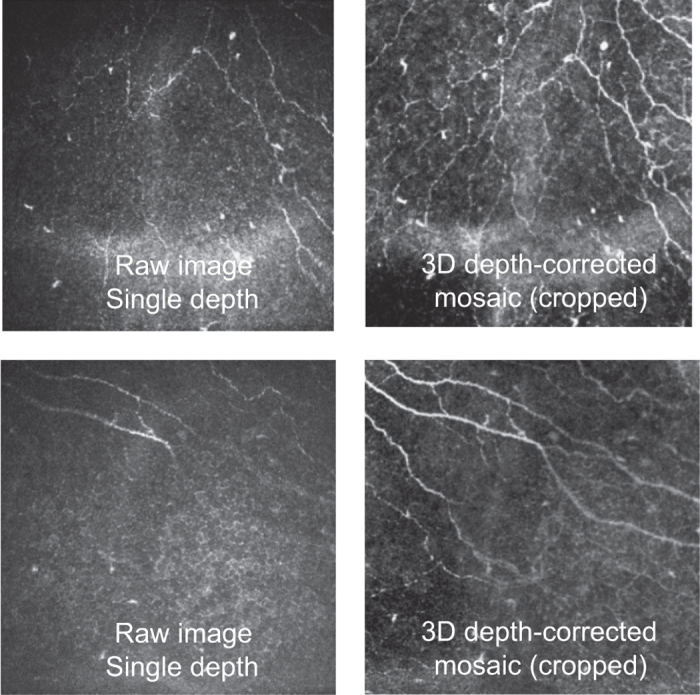
Mosaic quality improvement due to 3D SBP reconstruction. Nerve visibility in single, raw, non-depth-corrected images (left column) is poorer than corresponding regions from the mosaic dataset (right column).

Scenario 1 addresses the question of whether sampling a few IVCM images from the SBP (typically 2–8 are chosen) results in an accurate estimation of CNFL of the SBP. Scenario 2 reveals the magnitude of error if depth correction of the subbasal nerve paths in three dimensions is not employed, to indicate if raw 2D images can be used to simplify application of IVCM clinically. For these scenarios, 160 depth-corrected mosaics from the present dataset were deconstructed into component single fields-of-view, each representing a standard 400×400 μm area of the SBP. Where possible, up to 20 non-overlapping single fields were identified for each mosaic (Fig. 3a). In contrast to prior studies where partially overlapping fields^28^ or virtual fields of view^30^ have been used, the fields in the present study represented the actual locations of obtained single images with 0% overlap. At least 19 non-overlapping single fields could be isolated in 107 mosaics (Fig. 3b). The range of nerve density error (difference of mean CNFL based on sampling, from the reference mCNFL) for sampling 1 to 20 fields across 160 mosaics is illustrated graphically in Fig. 3c, for both scenarios (automated tracings shown; nearly identical results were obtained for manual tracing). For Scenario 1 with the best case of 20 non-overlapping depth-optimized fields, mean CNFL overestimated mCNFL by 10%. As fewer fields of view are used to sample the SBP, the magnitude of potential error in CNFL relative to mCNFL increases. For example, choosing 8 non-overlapping depth-corrected images results in a potential error range of −25% (underestimate) to 45% (overestimate), with exact error depending on the specific set of 8 images chosen. In most IVCM studies just 3 images are chosen for analysis of CNFL, resulting in potential error ranging from −50 to 65%. This however, is an optimistic estimate, as current practice does not utilize depth-correction of nerve paths. In the more common Scenario 2, multiple non-depth-corrected raw fields of view are sampled to determine CNFL (i.e., images available after clinical examination without further processing). In this scenario, the error curves skew towards an underestimation bias, due to substantially poorer visibility of nerves in non-depth-corrected images (Fig. 4). For the best case of 20 non-overlapping raw images, the resulting CNFL was on average 35% lower than mCNFL. As the number of sampled fields decreases, the magnitude of potential error increases. Sampling 8 non-overlapping images, CNFL error ranges from −60 to 0%, and for the common case of 3 non-overlapping images, CNFL error ranges from −80 to +20%.

### Phenotypic assessment of SBP architecture from mosaics

**Figure 5:**
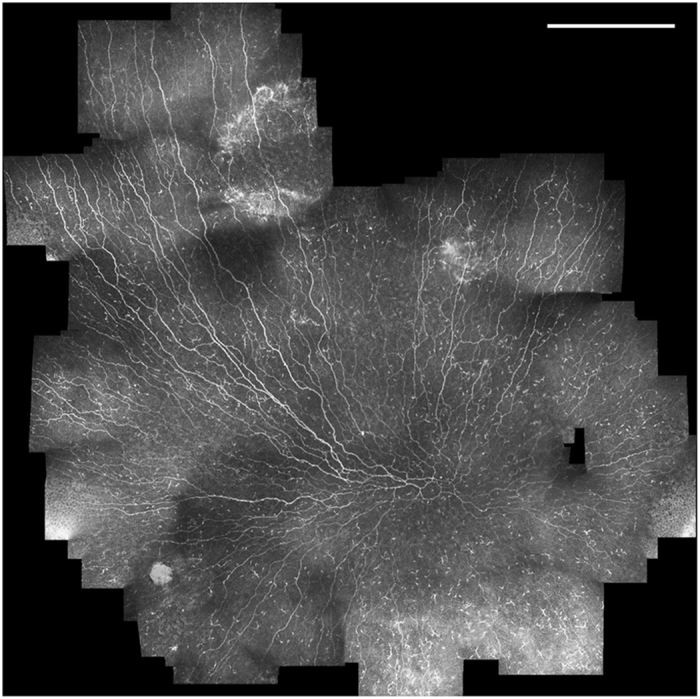
SBP mosaic from a subject in the examined cohort. A variable nerve thickness and reflectivity is present across the SBP, along with local areas of high and low nerve density. Nerve fiber paths have varying grades of tortuosity, particularly in the region of the inferocentral whorl. Dendritic cells are distributed throughout the SBP. Scale bar=0.5 mm.

An example of a wide-area depth-corrected SBP mosaic is presented in Fig. 5. Mosaics reveal variability of nerve features across the SBP. Local areas of high and low nerve density, reflectivity, width/reflectivity, and tortuosity are evident. The distribution of other features (penetration point of nerves through Bowman’s layer, lesions or discontinuities in the SBP, dendritic inflammatory cells, etc.) can also be assessed from the mosaics. Mosaics also enable investigation of global nerve architecture^33^ in the SBP in a manner not possible with single field of view IVCM images.

## Additional information

**How to cite this article:** Lagali, N. S. *et al.* Wide-field corneal subbasal nerve plexus mosaics in age-controlled healthy and type 2 diabetes populations. *Sci. Data* 5:180075 doi: 10.1038/sdata.2018.75 (2018).

**Publisher’s note:** Springer Nature remains neutral with regard to jurisdictional claims in published maps and institutional affiliations.

## Supplementary Material



## Figures and Tables

**Table 1:** Wide-field depth-corrected nerve mosaic characteristics, considering only the single largest mosaic per eye.

**Subjects**		**82**	
**Eyes**		**164**	
**Mosaics**		**163**	
	**Mean**±**SD**	**Min**	**Max**
Mosaic area (mm^2^)	5.95±1.8	1.45	11.26
Enhancement factor	37±11	9	70
No. of input nerve images	522±146	122	955
Mosaicking time (min)	106±60	6	348
Optimized mosaicking time (min)	7	0.75	15.63

**Table 2:** Parameters associated with the SBP mosaic dataset.

**Parameter**	**Explanation**
Image Name^a^	Filename of the image in the ‘Wide field SBP mosaics’ fileset.
Subject ID	Identification number of the subject in the cohort (subjects numbered from 1 to 128)
Eye	Image and clinical data corresponds to OS (left eye) or OD (right eye)
Mosaic area^a^	Area of the corneal SBP represented in the mosaic image (in μm^2^ )
Mosaic number	Identification appended to the end of the image filename to indicate a distinct mosaic lacking data to connect to other mosaics in the same eye, eg., m1, m2, etc. Most often the filename with ‘m1’ is the largest mosaic for the given eye.
Esthesiometry length	Length of nylon thread in Cochet-Bonnet esthesiometry, threshold for stimulation of blink reflex (in cm)
mCNFL (manual)^a^	Corneal subbasal nerve fiber length density across the entire mosaic (in mm/mm^2^), based on manual nerve tracing
Manual nerve tracing files	A separate fileset ‘Manual nerve tracing files’ has been included in the Figshare Collection, providing the raw manual nerve tracings for 182 mosaics (including all largest mosaics per eye). The fileset of 182 files in.ndf format is provided, indexed by Subject ID, Eye, and Mosaic number. The.ndf format can be read and analyzed within ImageJ/NeuronJ open source software. mCNFL (manual) has been calculated using these.ndf files.
mCNFL (auto)^a^	Corneal subbasal nerve fiber length density across the entire mosaic (in mm/mm^2^), based on an automated algorithm for nerve tracing
wCNFL (manual)^a^	Corneal subbasal nerve fiber length density in the whorl region defined by a 800 μm diameter circle centered on the corneal apex (in mm/mm^2^), based on manual nerve tracing
wCNFL (auto)^a^	Corneal subbasal nerve fiber length density in the whorl region defined by a 800 μm diameter circle centered on the corneal apex (in mm/mm^2^), based on an automated algorithm for nerve tracing
Subject group	Group membership of each subject. NGT=normal glucose tolerance, IGT=impaired glucose tolerance, <10=type 2 diabetes diagnosed less than 10 years prior to imaging, 10+=type 2 diabetes diagnosed 10 or more years prior to imaging
Diabetes duration	Years elapsed between diagnosis of type 2 diabetes and eye imaging examination. For subjects diagnosed less than one year prior to eye examination, ‘Newly diagnosed’ is indicated.
Raw nerve plexus images	A fileset ‘Raw nerve plexus images’ has been included in the Figshare Collection, to provide all the raw SBP images obtained by IVCM. These are all the raw image data used to assemble the mosaic images. The raw data consists of two compressed ZIP archive folders, the first containing raw image data for Subject ID numbers 1–60, and the second Subject ID numbers 61–128. When uncompressed, the archive consists of one folder per Subject ID. Within the folder are two folders for left (OS) and right (OD) eyes of that subject. Within each of these folders are the set of raw SBP images (typically several hundred images per eye).

^a^indicates that only the data for the largest mosaic image from each examined eye is available. Additional, smaller mosaic images from a given eye may be provided in the dataset, but image area and nerve parameters were not computed for the smaller mosaics.

**Table 3:** Clinical and demographic data associated with the cohort of examined subjects.

**Parameter**	**Explanation**
Subject ID	Identification number of the subject in the cohort (subjects numbered from 1 to 128)
Age	Subject age in years at time of eye imaging
Sex	M=male, F=female
Smoker	Smoking status at time of eye imaging, 1=smoker, 0=nonsmoker
HbA1c	Blood HbA1c level in mmol/mol
BMI	Body mass index in kg/m^2^
Avg fasting plasma glucose	Average fasting plasma glucose level in mmol/l. Data acquired only for those without confirmed type 2 diabetes.
Avg 2 h plasma glucose	Average 2-hour plasma glucose level in mmol/l. Data acquired only for those without confirmed type 2 diabetes.
